# Detection of Structural Changes in Tachogram Series for the Diagnosis of Atrial Fibrillation Events

**DOI:** 10.1155/2013/373401

**Published:** 2013-04-18

**Authors:** Francesca Ieva, Anna Maria Paganoni, Paolo Zanini

**Affiliations:** Dipartimento di Matematica, Modellistica e Calcolo Scientifico (MOX), Politecnico di Milano, Via Bonardi 9, 20133 Milano, Italy

## Abstract

Atrial Fibrillation (AF) is the most common cardiac arrhythmia. It naturally tends to become a chronic condition, and chronic Atrial Fibrillation leads to an increase in the risk of death. The study of the electrocardiographic signal, and in particular of the tachogram series, is a usual and effective way to investigate the presence of Atrial Fibrillation and to detect when a single event starts and ends. This work presents a new statistical method to deal with the identification of Atrial Fibrillation events, based on the order identification of the ARIMA models used for describing the RR time series that characterize the different phases of AF (pre-, during, and post-AF). A simulation study is carried out in order to assess the performance of the proposed method. Moreover, an application to real data concerning patients affected by Atrial Fibrillation is presented and discussed. Since the proposed method looks at structural changes of ARIMA models fitted on the RR time series for the AF event with respect to the pre- and post-AF phases, it is able to identify starting and ending points of an AF event even when AF follows or comes before irregular heartbeat time slots.

## 1. Introduction

**Figure 1:**
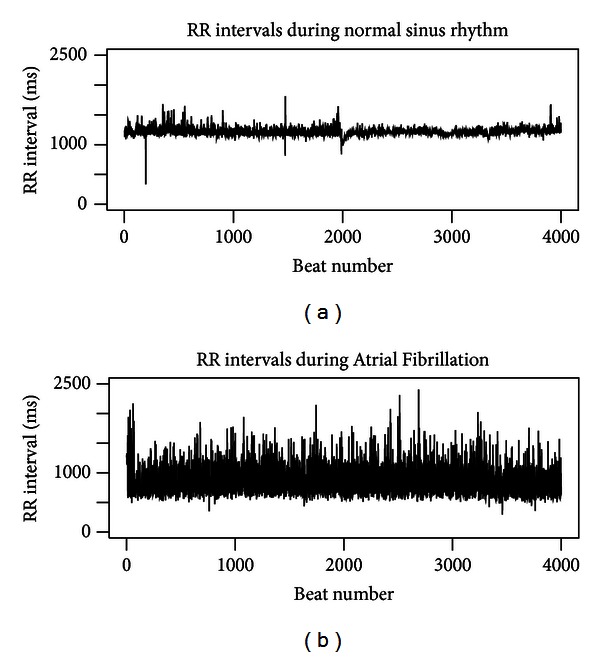
Typical series of RR intervals during normal sinus rhythm (a) and during Atrial Fibrillation (b).

During the last 20 years, there has been a widespread interest in the study of variations in the beat-to-beat timing of the heart, known as Heart Rate Variability (HRV) [1, 2]. This is due to several different reasons. HRV has been reported as strong predictor of cardiovascular mortality, and it is one of most popular parameter to assess the autonomic tone (see [3] and the references therein for a detailed discussion). Moreover, it represents a noninvasive way to assess postsurgical risks (see, e.g., [4]) or to investigate and tune gold standard practices [5]. Nevertheless, as highlighted in [6], the potential for HRV to be used widely in clinical practice remains to be established. When the sinus rhythm is normal, the tachogram series (i.e., the series of RR intervals; see Figure 1(a)) presents spontaneous beat-to-beat oscillations related to the autonomic nervous system regulatory action [7]. On the other hand, during arrhythmias, the spontaneous RR variability is perturbed and the spectral pattern changes according to the generating mechanisms of arrhythmia (see [8, 9]). Atrial Fibrillation (AF) is the most common cardiac arrhythmia, and involves the two upper chambers (atria) of the heart [10]. During AF, the normal electrical impulses generated by sinoatrial node are overwhelmed by disorganized electrical impulses that originate in the atria and pulmonary veins, leading to conduction of irregular impulses to the ventricles that generate the heartbeat. The result is an irregular heartbeat (see Figure 1(b)), which may occur in episodes lasting from minutes to weeks, or it could occur all the time for years. The natural tendency of AF is to become a chronic condition, and chronic AF leads to an increase in the risk of death.

**Figure 2:**
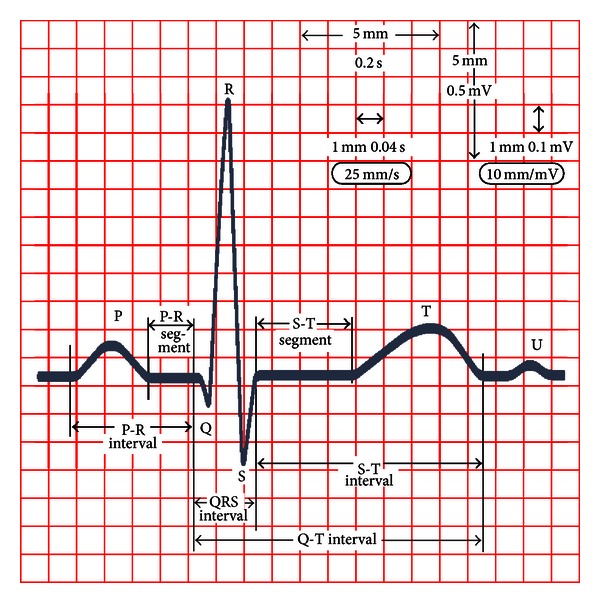
Stylized shape of a physiological single beat, recorded on ECG graph paper. Main relevant points, segments, and waves are highlighted.

The main device used in order to investigate the heartbeat is the Electrocardiogram (ECG) [11]. This diagnostic tool measures and records the electrical activity of the heart in details. The interpretation of these details allows for diagnosis of a wide range of heart diseases and AF among others. A stylized shape of an ECG is depicted in Figure 2. In general, atrial contraction shows up as the P wave; ventricular contraction is identified as a series of three waves, Q, R, and S, known as the QRS complex. The third wave in an ECG is the T wave. It reflects the electrical activity produced when the ventricles recharge for the next contraction, named repolarization (see [12] for more details on ECG).

Concerning the ECG detection of AF events, characteristic findings are the absence of P waves with unorganized electrical activity in their place and irregular RR intervals due to irregular conduction of impulses to the ventricles. While the analysis of P wave is quite complicate, the study of RR intervals is simpler. Hence, it could be an effective way to investigate the presence of AF and to detect when a single event starts and ends. Several examples exist in the literature (see [13–16]), which are focused on the peculiar variance of RR intervals during the AF process, and this variance is much greater than the one during the physiological heartbeat.

Anyway, in many situations, an AF event does not follow a physiological time slot but comes after other types of arrythmia. At the same time, in many cases, the irregular heartbeat does not disappear when the event finishes. According to these problems, it may be possible to look at an irregular heartbeat even when the AF event itself has not already started or has already finished. So, a method based on detection of changes in the variance of the process can lead to inaccurate results and can fail as described previously. Hence, methods which are not based on the analysis of the process variance are needed, in order to identify suitable quantities to characterize the different phases, say “pre- AF,” “AF,” and “post- AF.” To this aim, efforts are usually focused on changepoint detection of the spectrum or of the mean of a time series (see [17–20] among others). In these cases, the tachogram is considered as a time series (see [21, 22]), the order of the model is fixed, that is, orders *p*, *d*, and *q* of the autoregressive (AR), integrated (I), and moving average (MA) component, respectively, are previously established, and the focus is on the evolution of the estimated model parameters.

In this work, we assume that the tachogram, during an AF event, is characterized by a specific process. Hence we propose a different approach: we describe the phases of AF by means of ARIMA models characterized by different orders *p*, *d*, and *q*. The main issue becomes then to point out proper statistical methods for detecting changes in the order of the model. To achieve this goal, we firstly carry out a simulation study to test the new statistical method we propose, then we analyze data of 8 patients affected by AF. In particular we have for each patient the tachogram from two hours before to two hours after an event of AF. Although there are a lot of readings about the change point detection of time series, there is a lack of literature if the approach we just mentioned is considered.

 The paper is organized as follows. In Section 2, we introduce some elements of time series processes theory related to ARIMA models used for modeling the RR intervals time series. We present the statistical method developed for identifying the AF event (Section 2.3), based on the analysis of multiple test *P*-values with an improvement of the Bonferroni correction, and we test it in a simulation setting (Section 2.4), in order to assess the performance of the proposed method. Then in Section 3, we present the results obtained applying our method to real data (tachograms of patients affected by AF).  Section 4 contains discussion and conclusions.

All the simulations and the analyses of real data have been carried out using R statistical software [23].

## 2. Materials and Methods

In this section, we introduce ARIMA models [24] as a tool for modeling the RR time series dynamic. Then, we present the statistical techniques developed for identifying onset and end of AF events. Moreover, a simulation study is carried out to test the performance of the new method we propose, and results of simulations are discussed.

### 2.1. Autoregressive Integrated Moving Average (ARIMA) Models

Many empirical time series have no constant mean. Even so, they exhibit a sort of homogeneity in the sense that a suitable affine transformation could have constant mean. Models which describe such homogeneous nonstationary behavior can be obtained by supposing some suitable differences of the process to be stationary. Referring to the framework and theory treated in [24], we focus on the properties of the important class of models for which the *d*th difference (∇^*d*^ *z*_*t*_ = *z*_*t*_ − *z*_*t*−*d*_) is a stationary ARMA process. Then, let us consider the model

(1) $$\begin{array}{c} \phi \left(B\right)\nabla^{d}z_{t}=\theta \left(B\right)a_{t}, \end{array}$$

where *B* is the backward shift operator,

(2) $$\begin{array}{c} \phi \left(B\right)=1-\sum_{j=1}^{p}\phi_{j}B^{j},\;\;\;\theta \left(B\right)=1-\sum_{h=1}^{q}\theta_{h}B^{h}, \end{array}$$

with *ϕ*_*j*_, *j* = 1,…, *p* and *θ*_*h*_, *h* = 1,…, *q* suitable parameters to be estimated. Generally these estimates are performed through ML methods [24]. Process (1) is an Autoregressive Integrated Moving Average (ARIMA) process. If the autoregressive operator *ϕ*(*B*) in (1) is of order *p* and the moving average operator *θ*(*B*) is of order *q*, then (1) is an ARIMA(*p*, *d*, *q*) process.

### 2.2. Model Diagnostic Checking

Suppose to fit model (1) obtaining ML estimates ($\hat{\phi },\hat{\theta }$) for the parameters of interest. We will refer to the quantities

(3) $$\begin{array}{c} {\hat{a}}_{t}={\hat{\theta }}^{-1}\left(B\right)\hat{\phi }\left(B\right)\nabla^{d}z_{t} \end{array}$$

as the residuals. As the number of observations increases, ${\hat{a}}_{t}$ becomes closer to the white noise *a*_*t*_. Now suppose *p*, *d*, and *q* were chosen correctly and that we knew the true parameter values *ϕ* and *θ*. Then, the estimated autocorrelation *r*_*k*_(*a*) of the process *a* would be distributed approximately normally with zero mean (see [25]). Now, in practice, the parameters *p*, *d*, and *q* are unknown and only the estimates ($\hat{\phi },\hat{\theta }$) are available for calculating $\hat{a}$. Then, autocorrelation $r_{k}(\hat{a})$ of $\hat{a}$ can yield valuable evidence concerning the lack of fit. An interesting way to analyze the goodness of fit of the model is then to consider the $r_{k}(\hat{a})$ taken as a whole. Let us suppose that we have the first *K* autocorrelations $r_{k}(\hat{a})$ (*k* = 1,2,…, *K*) from any ARIMA(*p*, *d*, *q*) process. Then, it is possible to show (see [26]) that, if the fitted model is appropriate, the statistic

(4) $$\begin{array}{c} Q=\tilde{n}\left(\tilde{n}+2\right)\sum_{k=1}^{K}\frac{r_{k}^{2}\left(\hat{a}\right)}{\left(\tilde{n}-k\right)} \end{array}$$

is approximately distributed as *χ* ^2^(*K* − *p* − *q*), where $\tilde{n}\;=n-d$, with *n* equal to the number of observations. Therefore, an approximate test of the hypothesis of model adequacy may be performed. The statistic *Q* is called Ljung-Box statistic.

### 2.3. A Method to Detect Structural Changes in Time Series

 We now consider a phenomenon that evolves according to an ARIMA process. We wish to analyse a time series and to detect when such a phenomenon starts and/or ends. If this specific phenomenon is characterized by a higher (or lower) variability with respect to the current situation, then there is a huge number of methods effective in detecting these changes in variability. Examples are control charts (see [27]) and methods based on graphical analysis among others (see [15]). However, there are a lot of situations in which a phenomenon is not characterized by a modification of the variability, but by some changes in the process that generates the observations. In these cases, methods such those mentioned earlier are useless and other methodologies have to be considered. For example, in the literature, there is a huge quantity of methods that deal with structural changes in time series concerning changes of the mean or of the parameters values of the ARIMA model (see [19, 20] and the references therein). Nevertheless, we may be interested in dealing with a different situation. For example, we may consider a problem where the presence or the absence of a phenomenon is characterized not in a change of the parameters values of the model, but in a modification of the process itself. We wish to present here a method for dealing with this kind of situations.

As we mentioned before, our main goal is to identify the beginning and the end of a specific phenomenon modeled by an ARIMA process. This means firstly to identify the model parameters of the phenomenon under study, that is, the values of *d*, *p*, and *q*. As we have previously presented, in the case of a stationary model, the autocorrelation and partial autocorrelation function will quickly approach zero. Knowing that the estimated autocorrelation function tends to follow the behavior of the theoretical autocorrelation function, failure of this estimated function approaching zero rapidly might logically suggest that we should treat the underlying stochastic process as nonstationary in *z*_*t*_, but possibly as stationary in ∇^*d*^ *z*_*t*_. Once identifying one or more possible values for *d*, we move to the choice of *p* and *q*. This may be done considering the specific behaviors of the autocorrelation and partial autocorrelation functions and corresponding cut-off lags (see [24] for the details).

To identify the starting and ending times of the phenomenon of interest, we propose the following procedure. Consider the first *N* observations (with *N* much smaller then the number *n* of observations) and fit the identified model on this subsample. Then, the *P*-value of the Ljung-Box test (choosing a value for *K*) is recorded. These operations have to be repeated over the sub-sample from the second to the *N* + 1th observation. Once reaching the last observation, the procedure ends producing a “time series" of *P*-values which may be used to detect the beginning and the end of the phenomenon of interest.

The purpose is to test the null hypothesis that the phenomenon is present against the alternative hypothesis that the phenomenon is absent. This may be formalised as follows:

(5) H0:p=p¯∧d=d¯∧q=q¯  versusH1:p≠p¯∨d≠d¯∨q≠q¯,

where $\bar{p}$, $\bar{d}$, and $\bar{q}$ are the parameters indicating the order of the ARIMA process related to the phenomenon under study. In order to build the critical region for the test (5), the first *P*-values, say *M*, can be considered, and the rejection region can be constructed through a multiple test procedure, where the adjustment for multiplicity is based on the correction proposed by Simes. So doing, the approximate level of the test is equal to *α* (see [28] for the detailed work). The decisional criterion is the following. After the *M* *P*-values have been ordered from the minimum (say *p*_(1)_) to the maximum (say *p*_(*M*)_), the null hypothesis is rejected if for at least one *j* from 1 to *M* the following inequality is satisfied:

(6) $$\begin{array}{c} p_{\left(j\right)}\leq \frac{j\alpha }{M}. \end{array}$$

It can be proved that this procedure provides an approximate level equal to *α*. Furthermore the test results are less conservative than a test implemented using a classical Bonferroni correction, especially in this situation, where single tests are highly correlated.

 The method to detect start and/or end of a specific phenomenon follows these steps:

1. implement the test in (5)-(6) over the first *M* *P*-values, and at the *N* + *M* − 1th observation, the output is set to 0 if there is statistical evidence to reject the null hypothesis, while it is set at 1 otherwise;
2. repeat step (1) after a shift of one observation until the last one is reached.

Once the procedure ends, an output of 0′s and 1′s is available. Also, 1 indicates the presence of the phenomenon, 0 the absence. Starting and end points can be then detected through this last 0/1 time series.

### 2.4. Simulation Study

In order to validate the proposed method, different situations have been tested and analysed. The main goals are the following:

- to point out settings where our method performs at best,
- to assess the robustness of the method varying *α* and *N*,
- to make a sensitivity analysis over the parameter *K* of the Ljung-Box statistics.

The method presented in this paper is a technique to detect modification in the process underlying the observed phenomenon. We chose an ARIMA (0, 1, 1) as Reference Process (RP), considering a sequence of 7000 realizations from a process, say *P*_pre_, then 40000 realizations from the reference model, and finally 7000 realizations from another different process, say *P*_post_. For all the simulations, the value of *M* was fixed equal to 100. In this particular case, test (5) becomes.

(7) $$\begin{array}{c} H_{0}:p=0\wedge d=1\wedge q=1\;\text{versus}\;H_{1}:p\neq 0\vee d\neq 1\vee q\neq 1. \end{array}$$

We tested it in different situations; in the first, second, and third simulations, *P*_pre_ and *P*_post_ are very different from RP, whereas in the fourth one they are quite similar. In particular we set *P*_pre_≡*P*_post_, and we considered an ARIMA (4, 1, 2), ARIMA (5, 1, 3), ARIMA (2, 2, 0) and ARIMA (1, 1, 1), respectively. 

**Figure 3:**
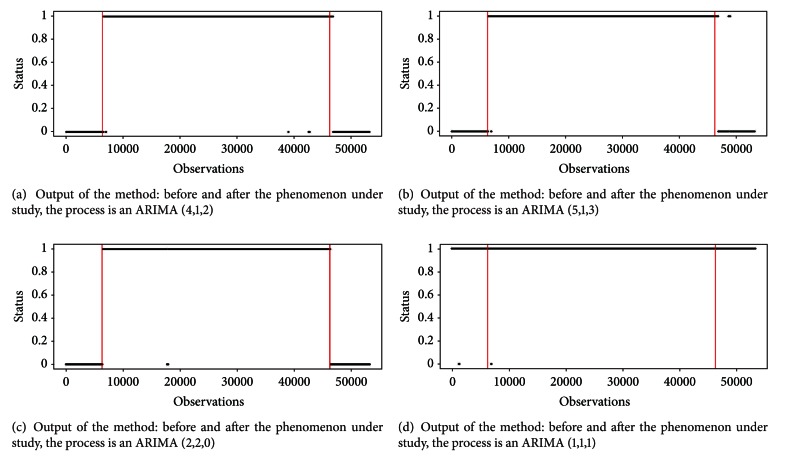
Analysis of the output of the method changing the process underlying the observations before and after the phenomenon. Red lines represent the start and the end of the phenomenon.

The parameters values for these simulations have been chosen randomly, under the constraint that the models were admissible. Their values are reported in Table 1. Figures 3(a), 3(b), and 3(c), obtained fixing *K* = 5, *N* = 600, and *α* = 0.01, show that our method works very well in the first 3 settings, where the correspondence among the real starting and end points (red lines) and the 0/1 sequence is visible. In the fourth simulation, instead, the method is less able to catch the phenomenon under study, as it is shown in Figure 3(d).

**Figure 4:**
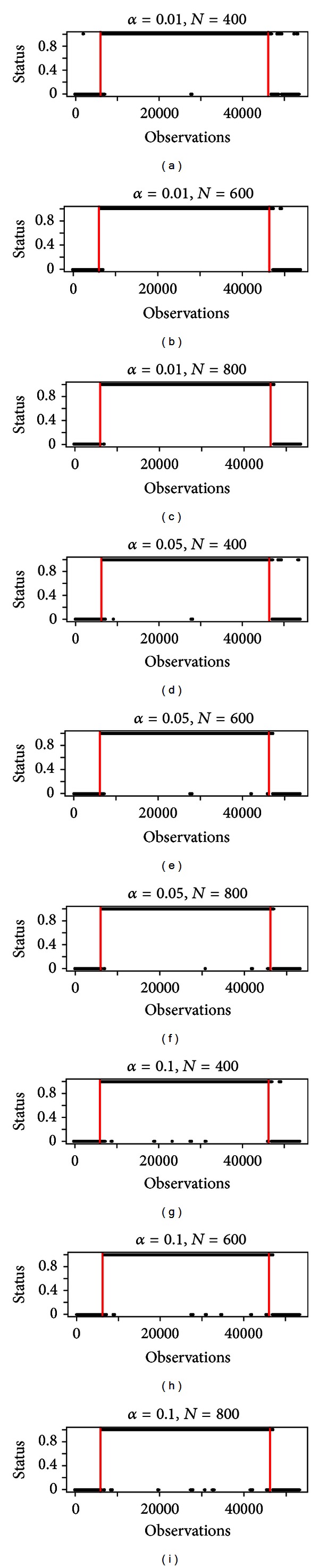
Output of the method varying *α* (along the rows) and *N* (along the columns).

In the following, we focus on the case related to Figure 3(b), where the generating process is an ARIMA (0, 1, 1), anticipated and followed by a process of observations generated from an ARIMA (5, 1, 3). Cases (3a) and (3c) give similar results. We analyse how the power of the test in (7) is affected by *α* and *N*. For this analysis, we considered *K* = 5. If *α* was the real probability of the type-I error, the power would increase as *α* grows. We do not have the real probability of the type-I-error, but only an upper estimate. Nevertheless, we would observe the power growing as *α* increases. Another parameter that affects the power of the test is *N*. Again, the bigger *N* is, the greater the power of Ljung-Box test is. So, also the power of the global test should be raised. In Figure 4, the output of the method varying *α* (along the rows) and *N* (along the columns) is shown. It can be inferred that the behavior of the method is consistent, since the number of errors before and after the phenomenon decreases as *α* and *N* increase, as we expected.

We consider, for different values of *α* and *N*, the empirical type-I error probability and the empirical power computed over 40 simulations.  Table 2 shows that this test is conservative, but the empirical type-I error probability is not so far from the nominal level of the test. Moreover, the results presented in Table 3 suggest that, once *α* is fixed, it is possible to increase the power of the test tuning *N* in a suitable way. Then, one could think to set a very high value of *N* in order to obtain a satisfactory power. However, this is not costless. In fact, the higher the value of *N* is the greater the delay in starting and ending points detection is. 

**Figure 5:**
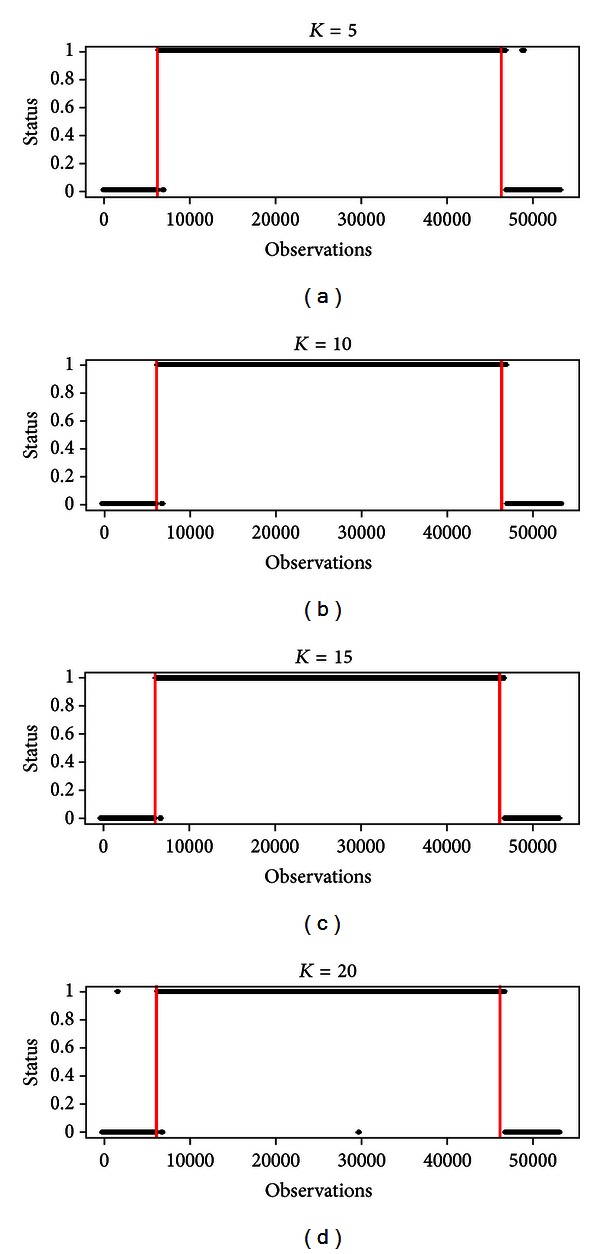
Output of the method varying *K*.

Hence, the choice of the parameter *N* is regulated by a tradeoff between the desired power of the test and the delay in the detection of the phenomenon. To conclude the simulations' analysis, we would like to infer about the parameter *K* of the Ljung-Box statistics in order to understand if the method is affected by a modification of its value. Let consider the situation where observations before and after the phenomenon were generated by an ARIMA (5, 1, 3), and fix *α* = 0.01 and *N* = 600. In Figure 5, the output of the method for different values of *K* (equal to 5, 10, 15, and 20, resp.) is shown. Although the outputs are different, no pattern of dependence on *K* appears.

## 3. Results and Discussion

Let us consider now an application of the method proposed in this paper to real data. Specifically we analysed RR intervals of 8 patients during Atrial Fibrillation (AF).

Data have been supplied to authors by Professor Luca Mainardi responsible of the Biomedical Signal Processing Laboratory of the Department of Bioengineering, Politecnico di Milano. Before patients underwent an ablation intervention, a seven-day Holter trace had been recorded using a one-channel *Del Mar Reynolds* Holter recorder, with sample frequency equal to 128 Hz. This protocol of data collection is in accordance with the Declaration of Helsinki for research with human beings. The data available are the RR intervals of such patients from two hours before to two hours after an event of AF. The duration of the phenomenon is different between patients and it is displayed in Table 4. 

**Figure 6:**
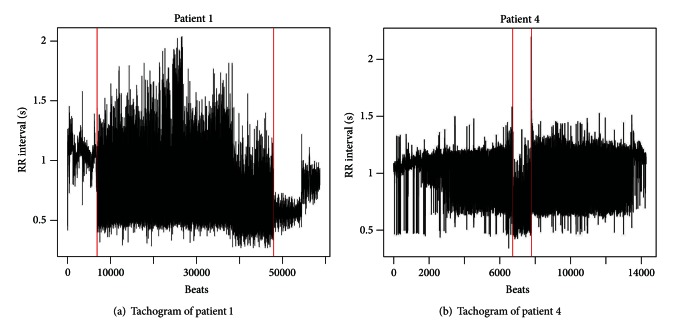
Tachogram of two patients. For patient 1 (a), AF event comes after and is followed by normal sinus rhythm, characterized by low heart rate variability. Patient 4 (b) presents a high rate variability even before and after the AF event.

We want to detect the event of AF from the study of tachogram series. In some cases, the variability of RR intervals during AF is very high with respect to the physiological heartbeat. However, this remarkable change in the variability of the phenomenon could be absent, as highlighted in Figure 6. This is an example where the traditional methods based on detection of changes in the process variability are ineffective in detecting AF starting point.

**Figure 7:**
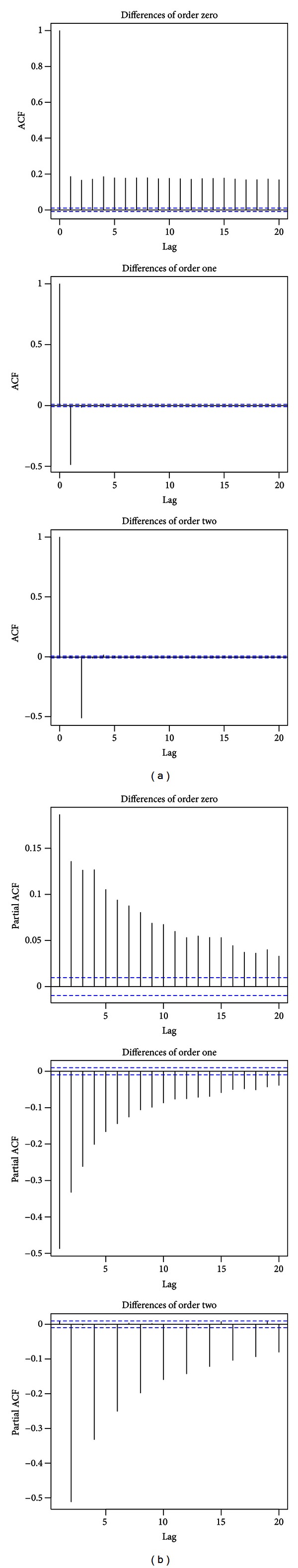
Patient 1: autocorrelation (a) and partial autocorrelation (b) functions for the time series of RR intervals, of the differences of order one and of the differences of order two.

The first step consists in the identification of a model for the RR intervals during AF. We used the autocorrelation and partial autocorrelation functions to determine a suitable model. As it is shown in Figure 7, the autocorrelation function of ∇*z*_*t*_ is truncated after the lag number one, while that of ∇^2^ *z*_*t*_ is zero after the lag two. This behavior is typical of an ARIMA (0, 1, 1) and (0, 2, 2). Then, we set RP  *≡*  ARIMA (0, 1, 1). The same analysis done on the RR time series of pre-and post-AF does not lead to the same conclusions. Indeed autocorrelation and partial autocorrelation functions do not highlight these characteristics. Hence, the assumption that during Atrial Fibrillation the stochastic process generating the RR time series is different from the one that models other phases seems reasonable. Then, we would like to analyse the performance of the method in detecting start and end of such a phenomenon. 

**Figure 8:**
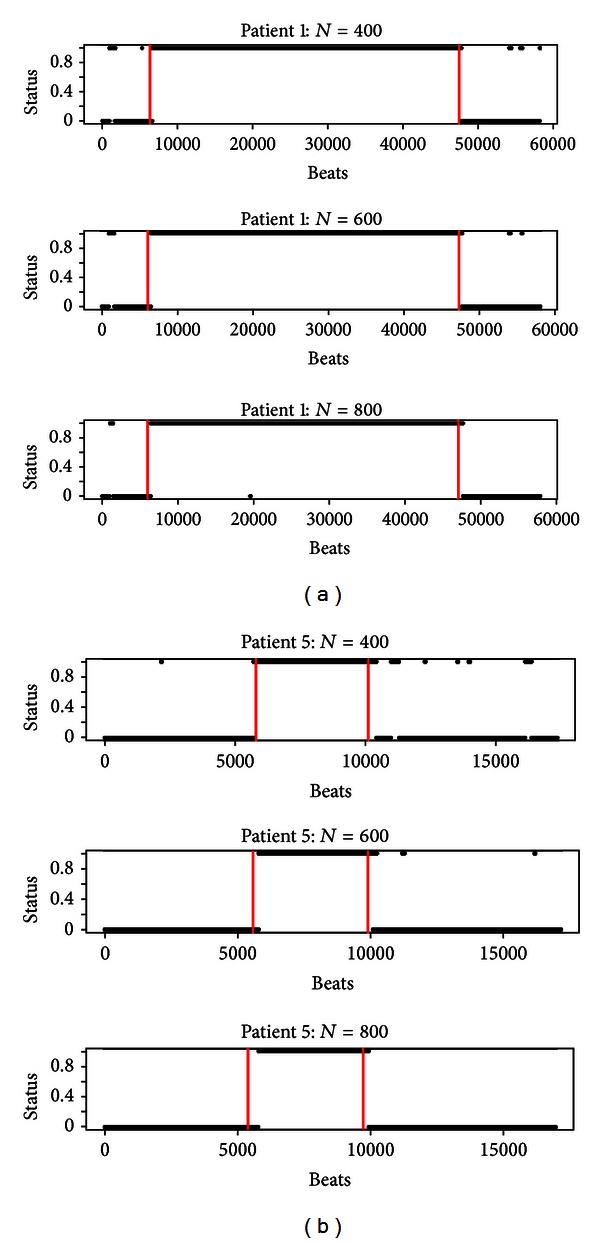
Output of the method for the patients 1 (a) and 5 (b) varying *N*.

In order to achieve this goal, let us fix the following values for parameters: *K* = 5, *α* = 0.001, and *M* = 100. Since in Section 2.4 the parameter *N* has been highlighted as the most important in affecting the performance of the proposed method, we analyse the output as *N* varies. In Figure 8, the outputs of the method applied to patients 1 and 5 are shown. We present here only the output for two patients, because the results for the other patients are quite similar.

Some considerations can be extrapolated observing Figure 8. First, we may point out to the behavior of the method as *N* increases. It can be seen that this behavior is in agreement with conclusions drawn from simulations. Then, we may analyse the delay in the detection of start and end of AF and the number of errors.

Dealing with the delay, since each observation is the time between an R peak and the following one, we can evaluate the time of the delay in the detection of the event of AF and not only the number of observations. As it is shown in Table 5, the delay in detecting the phenomenon is negligible if compared with the duration of AF, except for patient 4 affected by a very short AF event. Moreover, in some cases, the method is able to detect the AF event in advance. 

Another important point we want to focus on is the number of errors made by the proposed method. From a first insight of Figure 8, we can observe that the most part of the errors seems to involve a few number of consecutive observations. 

Then, a correction can be implemented in order to reduce the number of errors (in this case, the whole time interval detected in a wrong way is considered as an error). We introduced an artificial time delay: after the first instant of output switching from zero to one (or vice versa), we wait for a given time to declare the AF event started (or ended); only if after this time the method is still indicating the presence (or absence) of the phenomenon, we can detect it. The introduction of this correction and its duration are problem driven. Since AF is not a dead risk pathology, the problem concerning the number of errors is more important than the detection delay, and so we chose to insert an artificial time delay of 3 minutes. Doing that, we decreased considerably the number of errors, as shown in Table 6. 

## 4. Conclusions

In this paper, we proposed a statistical tool to identify starting and ending points of an event of AF (a common cardiac arrhythmia characterized by an irregular heartbeat) starting from the analysis of the RR intervals series. We presented a method based on time series analysis, and we performed a statistical test to automatically recognize the phases “pre-AF,” “AF,” and “post AF,” especially in those situations where the AF event does not follow a physiological time slot and/or the irregular heartbeat is still present when the event finishes. The novelty of this work consists in looking at a structural change of the order (*p*, *d*, or *q*) of ARIMA model fitted on the RR time series for the AF event with respect to the “pre-AF” and “post-AF” phases. We tested the proposed method on different simulated data, taking a reference ARIMA model for the AF phase, and varying the model of “pre-AF” and “post-AF” phases.

Then, we applied the method to real RR intervals data. The results we obtained confirmed the goodness of the proposed method, which seems to be able to identify starting and ending points of an event of AF even when AF follows or comes before irregular heartbeat time slots. This is the innovative feature of our method, because the large variety of techniques that deal with the detection of AF do not take into account this particular situation. Since our method analyzes structural changes of the order of the ARIMA model, it can detect AF episodes also in those particular cases when before and/or after the AF event the heartbeat does not follow a normal sinus rhythm, characterized by a significative lower variability. This fact confirms that this methodology may become a helpful tool for the online and/or offline detection of AF. In particular this method could be useful in an offline control of Atrial Fibrillation events, such as a Holter monitor that is a prolonged type of ECG tracing. Since the traditional detection of AF through the analysis of the P wave might be long and hard and, in general, it is simpler to extract the RR intervals from a Holter, the proposed method could represent an automatic diagnostic tool that simplifies the detection of AF events.

## Figures and Tables

**Table 1:** Parameters values used in the simulations. The first four models refer to *P*_pre_ and *P*_post_, while the fifth model refers to RP.

ARIMA	*ϕ*_1_	*ϕ*_2_	*ϕ*_3_	*ϕ*_4_	*ϕ*_5_	*θ*_1_	*θ*_2_	*θ*_3_
(4,1,2)	0.52	0.35	−0.04	0.11	/	−0.07	0.12	/
(5,1,3)	−0.66	−0.3	0.24	0.01	0.14	−0.08	−0.19	−0.29
(2,2,0)	−0.08	−0.25	/	/	/	/	/	/
(1,1,1)	−0.15	/	/	/	/	0.12	/	/
(0,1,1)	/	/	/	/	/	0.3	/	/

**Table 2:** Empirical type-I error probability varying *N* and the nominal value *α*.

	*N* = 400	*N* = 600	*N* = 800
α = 0.01	0.004547	0.005300	0.004969
α = 0.05	0.025889	0.028244	0.027377
α = 0.1	0.051221	0.055458	0.058005

**Table 3:** Empirical power varying *N* and the nominal value *α*.

	*N* = 400	*N* = 600	*N* = 800
α = 0.01	0.676305	0.873391	0.960046
α = 0.05	0.828791	0.945367	0.987309
α = 0.1	0.880587	0.967746	0.993346

**Table 4:** Duration and number of beats of the event of AF.

Pat. no.	Duration AF (min.)	Beats
1	521	41085
2	613	43178
3	433	52937
4	13	1066
5	56	4326
6	442	52661
7	319	28229
8	229	17989

**Table:** (a) Delays detecting the start of AF

Pat. num.	*N* = 400 (min.)	*N* = 600 (min.)	*N* = 800 (min.)
1	4.3	4.9	5.4
2	4.5	6.1	7.3
3	−2.4	0.2	−4.6
4	3.9	5.9	8.4
5	−1.4	2.7	5.6
6	−2.2	1	2.8
7	16.6	16.8	29.1
8	4.8	6.1	7.5

**Table:** (b) Delays detecting the end of AF

Pat. num.	*N* = 400 (min.)	*N* = 600 (min.)	*N* = 800 (min.)
1	3.2	4.6	6
2	5.6	8.5	12
3	6.8	7.2	8.9
4	7.3	9.8	10.1
5	5.1	5	3.3
6	−3.3	−6.9	−6.3
7	3.2	5.3	7
8	3.3	5.3	7.2

**Table 6:** Number of errors before (bef. corr.) and after (aft. corr.) the introduction of the artificial time delay (we fixed *N* = 600).

Pat. no.	Type-I errors (bef. corr.)	Type-I errors (aft. corr.)	Type-II errors (bef. corr.)	Type-II errors (after corr.)	Duration AF (min.)
1	0	0	4	1	521
2	1	0	0	0	613
3	16	6	1	1	433
4	0	0	4	4	13
5	1	0	2	0	56
6	23	5	3	1	442
7	8	1	8	3	319
8	0	0	10	6	229

Total	49	12	32	16	
